# Dual optical force plate for time resolved measurement of forces and pressure distributions beneath shoes and feet

**DOI:** 10.1038/s41598-019-45287-9

**Published:** 2019-06-20

**Authors:** Christopher G. Tompkins, James S. Sharp

**Affiliations:** School of Physics and Astronomy, University of Nottingham, University Park, Nottingham, NG7 2RD UK

**Keywords:** Polymers, Applied physics, Optical physics, Imaging techniques

## Abstract

Frustrated total internal reflection (FTIR) imaging was used to perform remote optical measurements of the forces/pressures exerted beneath shoes and feet during a number of different training activities including countermovement jumps, jogging and drop jumps. A single camera was used to simultaneously image two acrylic, FTIR waveguide imaging elements from below, at frame rates up to 200 frames per second. The images obtained using the camera were converted into pressure/force maps using a previously developed theory which combines the mechanics of contact of soft objects and the scattering of evanescent waves. The forces obtained from the optical measurements were shown to be in good agreement with measurements obtained from load cells placed beneath the FTIR imaging elements. The ability to produce accurate spatial maps of the force/pressure distribution beneath soft contacting objects such as feet and shoe outsoles at high frame rates has numerous potential applications in sports sciences and medicine.

## Introduction

Measurement of the forces exerted beneath soft contacting objects such as shoes and human feet have a number of applications in sports science & engineering and medicine. For example, the measurement of force/pressure distributions beneath the feet of athletes during routine training activities can be important in ensuring that they distribute their weight uniformly and develop physical strength in both legs evenly^1^. Similar measurements are also helpful for the design and optimisation of sports shoes, where the size, placement and orientation of structures on a shoe outsole can be used to improve characteristics such as impact resistance^2^ and frictional interactions^3^. Medical applications include measuring the pressure distributions beneath the feet of patients with spina bifida^4^ and diabetic patients. In the latter case, these measurements are used to locate and facilitate treatment of painful ulcers whose early detection can prevent the need for whole foot amputation^5^. Pressure measurements are also valuable in monitoring progress during the rehabilitation of athletes^6^ or elderly patients who have recently suffered a fall^7^, and in the design of orthotic^8^ and prosthetic devices^9^.

Conventional pressure mats are typically based upon an array of electrically addressable elements comprising resistive, capacitive or piezoelectric sensors that are sensitive to the local pressure that is exerted upon them^10^. These devices tend to be quite expensive and inaccessible to many researchers and clinics. However, optical methods of force detection offer a potential low-cost alternative to electrically addressable pressure mats. One such technique involves the use of frustrated total internal reflection (FTIR) to measure the amount of light that is scattered in the regions of contact between a soft object and a hard transparent slab of material (often acrylic or glass). Under normal operation, light is totally internally reflected inside the slab of material by careful design of the illumination conditions. The geometry of the device is chosen in such a way that light bouncing around inside the slab is always incident on the slab-air interface at an angle that is greater than the critical angle for total internal reflection^11^. In this way, the light remains confined inside the slab and produces a waveguide element^11,12^.

When a soft object is brought into conformal contact with the surface of the waveguide, the optical boundary conditions at the interface can change and it is possible for light to escape. This frustration of the total internal reflection condition occurs as a result of refractive index matching at the interface and/or due to the presence of scatters that are placed close to the interface^11^. When light escaping from the wave guiding FTIR element interacts with scattering objects (e.g. pigments, dyes, colloidal particles) within the material, the scattered light can be detected using a suitable camera and even by eye (if the scattered radiation is at visible wavelengths). Scattering of light only occurs in the regions where the contacting object is within a wavelength or so of the surface of the waveguide. This makes the technique highly surface sensitive and particularly useful for imaging the regions of contact between different materials. This has enabled the predictions of detailed theories of the mechanics of contact between objects to be tested^13^.

Betts and Duckworth^5,14,15^ were among the first to discover that the intensity of light scattered at a waveguide surface increases with the load applied to a soft contacting object. These authors used video tape to record the pressure distributions beneath the feet of diabetic patients. At the time, machine vision technology was not particularly advanced and computing power was still relatively expensive. This made the processing of FTIR images difficult.

High-speed cameras and computers are now relatively inexpensive and this has made the technology more accessible. Since the pioneering work of Betts and Duckworth and others, FTIR imaging has been used in a wide range of applications including measuring the area of contact and forces between microscopic objects^16,17^, rough surfaces^13^ and soft solids^11^. It has also found application in the measurement of the gait of small animals^18,19^ and insects^20^ as well as in the development of touch sensitive controllers^21^ and in the measurement of pressure beneath tyres^22^. Some of the more recent studies using this technology include imaging of shoes for forensic purposes^12^ and fingerprint imaging for biometric applications^23^.

A detailed mathematical treatment of the FTIR light scattering phenomenon had not been provided until recently. Sharp *et al*. derived a combined light scattering and contact mechanics theory to explain the dependence of the measured scattered light intensity on the mechanical, optical and surface properties of the contacting material^11^. A simple analysis which considers only the near surface strains in the sample predicts that the scattered light intensity, *I*_*pp*_, detected by a single pixel on an imaging camera has the form^11^;

1 $${I}_{pp}=\frac{{\pi }^{4}{A}_{p}{I}_{o}{\alpha }^{2}(1+co{s}^{2}{\theta }_{s}){\varphi }_{o}}{2{\lambda }^{3}{D}^{2}{n}_{o}cos{\theta }_{r}}{[\frac{3(1-{\nu }^{2})P}{E}]}^{\frac{2}{3}}$$

where *P* is the applied pressure, *I*_*o*_ is the incident intensity on the waveguide surface, *λ* is the wavelength of light being scattered, *A*_*p*_ the area imaged by a pixel, *n*_o_ the refractive index (real part) of the contacting material and D the distance between the camera and the contact regions being imaged. *θ*s is the scattering angle (≈90° for the geometry used here) and $cos{\theta }_{r}={(1-{(\frac{{n}_{w}}{{n}_{o}})}^{2}si{n}^{2}{\theta }_{s})}^{1/2}$ where *n*_*w*_ is the refractive index of the waveguide material. The parameters *E*, *ϕ*_o_ and *α* are the Young’s modulus of the contacting material and the concentration and volume polarizability of the scatterers within it respectively.

Sharp *et al*. also developed a detailed light scattering theory which gave a more in depth treatment of the strain distribution within a contacting object^11^. This resulted in predictions for the light scattering intensity which agreed well with the data over an extended range of applied pressures. It was shown that the data obtained over the full range of loading could be approximated to a one third power law scaling of the scattering intensity on the applied pressure (instead of the two third power law scaling predicted by equation 1). This was shown to agree well with data obtained from samples with a range of different geometries (e.g. flat punches, hemispherical indenters and shoe outsoles), elastic moduli, surface roughness values, colours and pigment/scatterer concentrations.

The work performed by Sharp *et al*.^11^ is extended here to the application of measuring the evolving pressure distribution beneath human feet and shoe soles of different colours, mechanical properties and with different tread patterns. FTIR imaging measurements were collected by using a single camera to image two platforms simultaneously. Image processing techniques were then used to analyse the response of each platform separately and to extract the pressure distributions beneath the contacting shoe outsoles and feet.

During this work, subjects were asked to perform a number of relatively straight-forward training activities (including jogging on the spot, countermovement jumps and drop jumps) and FTIR images were collected as they interacted with the platforms. Pressure maps were extracted from the FTIR images and the total forces obtained by summing the pressures in the images were compared to load cell measurements obtained from each platform. We demonstrate that the data obtained using this device is comparable to that collected from simple force platforms (which measure the total force exerted) with the additional capability of providing spatially resolved information about the changing pressure distribution beneath the shoes/feet.

## Experimental

### Construction of the dual platform FTIR imaging device

**Figure 1:**
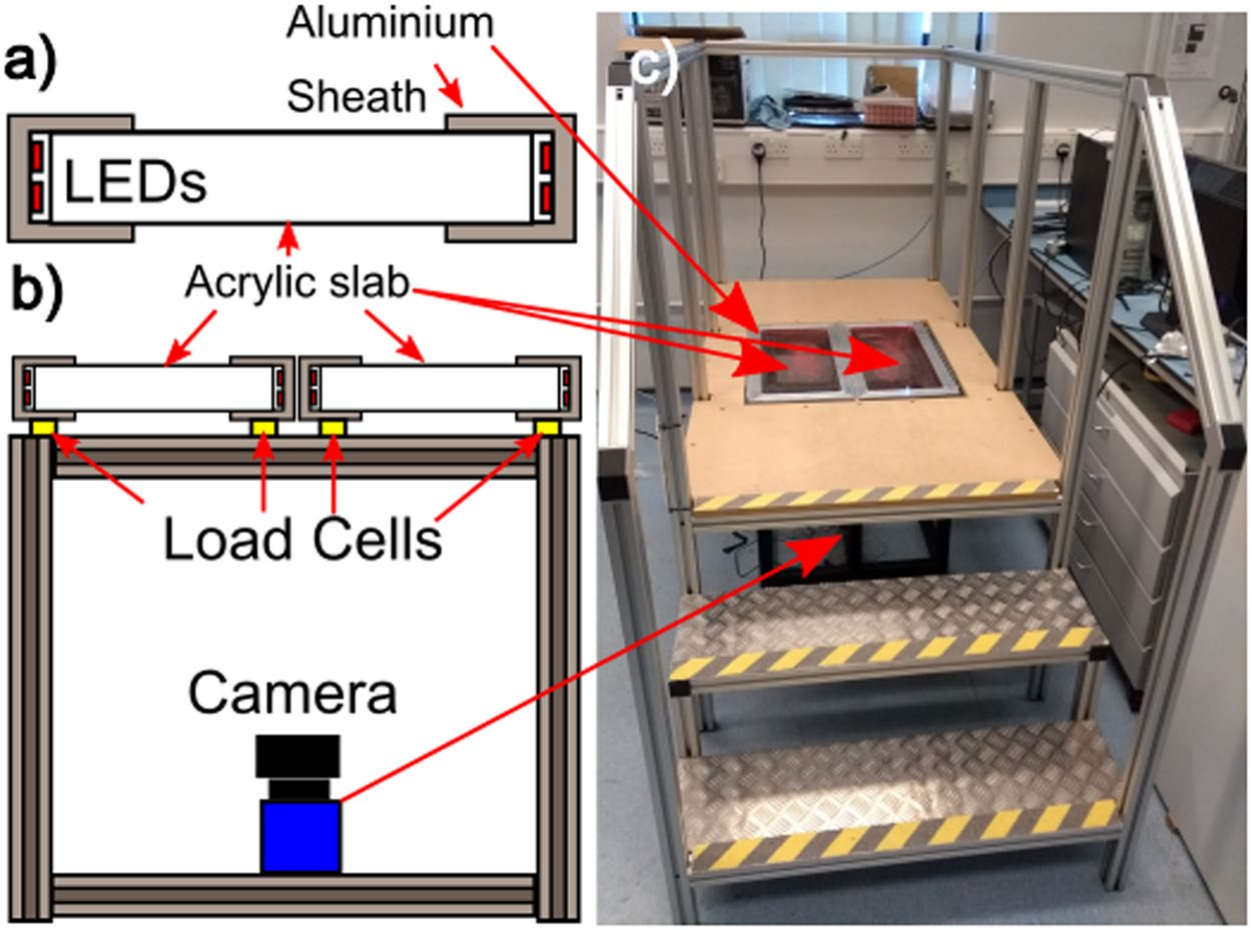
Construction of the dual platform imaging device. (**a**) Acrylic slabs were wrapped with ultrabright red LEDs and covered in an aluminium sheath to produce FTIR imaging elements. (**b**) Two of these slabs were mounted on a sturdy aluminium frame with load cells directly beneath them. A single camera was used to image the top surface of both platforms simultaneously. (**c**) The entire platform was mounted inside a larger, sturdy platform equipped with steps and handrails.

The dual imaging platform was constructed by wrapping strips of ultra-bright red LEDs (wavelength 632 nm) around the outside edges of two 600 mm × 300 mm × 25 mm thick slabs of transparent acrylic polymer. The LEDs were held in place by an aluminium sheath that also served to mask part of the flat faces of the acrylic slabs to ensure proper waveguiding of the light generated by the LEDs (Fig. 1a). Masking the flat faces in this way ensured that only light that is incident on the acrylic-air interface at an angle, *θ*, greater than that required for total internal reflection (*θ*_*c*_, ~42° for the acrylic-air interface) was allowed to propagate inside the slabs. Under these conditions the light remains confined inside the acrylic slab until an object (such as a shoe outsole or a foot) is brought into intimate contact with it. When this occurs, the boundary conditions experienced by the light confined inside the slab change and it is able to interact with the outsole or foot, but only in the regions of contact. If the object being brought into contact is capable of scattering light of the wavelength confined inside the slab, then a camera can be used to observe this scattering. In this and previous work, it was shown that the intensity of light scattered by a contacting object increases with the applied load/pressure that is exerted in the contact regions. In this way, a camera placed beneath the slabs was used to image the spatial variations in scattered intensity and extract information about the varying pressure distribution beneath the contacting objects. This was done using a method reportedly previously^11^ and which is explained briefly below.

A Basler AC800-510uc USB camera was placed beneath the imaging platform, connected to a PC running software written in LabVieW (National Instruments) and used to image the top surface of both acrylic slabs simultaneously. The slabs were each mounted on separate load cells so that independent measurements of the total force exerted on their surfaces could be obtained (Fig. 1b). These force measurements were collected electrically by detecting changes in the resistance of the load cells using a simple bridge circuit connected to a home built instrumentation amplifier that was in turn connected to a USB-6008 (National Instruments) data acquisition card and the same PC used to acquire images from the camera. Load cell measurements were acquired using a sampling rate of 200 Hz in all experiments.

Calibration of the pressure dependence of the scattered intensity response was performed for each of the slabs. This was achieved by using the camera to measure the scattered light intensity when flat objects were pressed into contact with the waveguide surface under varying loads^11^. Having calibrated the intensity-pressure response of the waveguide, high-speed imaging with inline image processing was then used to obtain spatially resolved information about the pressure distribution beneath contacting objects. This was achieved with a time resolution of ~0.005 seconds (200 frames per second) and a spatial resolution of 0.62 mm × 0.62 mm i.e. the size of the region imaged by a single pixel.

The entire assembly was mounted on a sturdy aluminium frame and a larger platform built around it (complete with steps and handrails) to facilitate ease of access (Fig. 1c). At this point it is worth noting that the same aluminium frame containing the imaging platform could have been sunk into a suitably sized pit in the ground. In this case, the larger platform was built to avoid digging holes in the laboratory floor and to allow the imaging platform to be moved if required.

### Image analysis and the construction of pressure distribution maps

**Figure 2:**
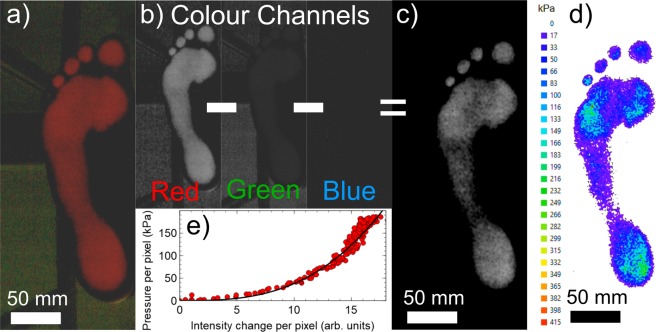
FTIR Image analysis. Colour images collected using the camera (panel a) are split into the corresponding red green and blue colour channels (panel b). The green and blue channels are subtracted from the red channel to remove the background to give the grayscale image in panel c). The grayscale image is then converted to the pressure map in panel d) using a pressure-intensity calibration curve similar to that shown in panel e). The solid line in panel e) shows a simple power law fit to the calibration data.

Colour images acquired using the USB camera were processed in order to extract the pressure distribution beneath the contacting objects used in this study (Fig. 2a). Briefly, each image was split into separate colour channels (red, green and blue). The blue and green channels were then subtracted from the red channel (Fig. 2b). This process has the effect of removing all background artefacts created by external light sources and retaining information about the intensity of the light scattered by regions of contact^11^. The background subtraction process resulted in the production of 8-bit grayscale images with pixels that each take a value between 0 and 255 - dependent upon the intensity of scattered light detected by the camera (Fig. 2c).

Individual pixel intensities were converted into the equivalent pressures (Fig. 2d) using a fit to a pressure-pixel intensity calibration curve, similar to that shown in Fig. 2e. The cubic power law relationship between the applied pressure and scattered light intensity shown in this panel results in uncertainties in the pressure values obtained from individual pixels of between 5 and 10%. These uncertainties are determined mainly by the bit depth of the camera being used and by noise in the intensity measurements. In all of the experiments that are reported here, the uncertainties in the total forces measured using the load cells were +/−5 N. Uncertainties in the optically derived total forces were typically higher and were in the range +/−10 N to +/−40 N range depending upon the scattering response of the objects at the wavelength used to excite the imaging platforms (632 nm). Objects that scattered well at this wavelength (e.g. red objects) gave optical measurements of the force with lower uncertainties, while objects that scattered the light more weakly (e.g. blue objects) gave rise to larger uncertainties.

Calibration curves obtained for different contacting materials have been shown to have a similar shape to that shown in Fig. 2e. However, the intensity of light scattered by materials with different colours will be different. Sharp *et al*.^11^ have shown previously that differences in the colour of the object can be corrected for by applying a material dependent scaling factor to the scattered intensity in order to obtain the correct force/pressure. In the present study, the scaling factor was determined automatically by the software for each new material studied. This was done by determining the scaling factor which gave the best fit between the measured load cell force and the total force derived from summing the pressures in the FTIR images over the course of the experiment. Once this simple calibration had been completed for the first experiment and the scaling factor determined using a particular subject, the same material could be used to perform additional experiments without the need for the load cell measurements. However, the load cell measurements were collected in every experiment to ensure the reliability of the optical measurements. Measurements were repeated three times for each subject to check that the force measurements, the optical force calibrations and the associated uncertainties were reproducible for each experiment being performed.

It is worth stressing that the calibrations are dependent only on the material properties of the contacting object. Hence, if different subjects were to wear the same shoe, the calibrations for the shoe material would remain valid for experiments performed on all the subjects. However, small differences in the properties (mechanical and optical) of the soles of feet between different individuals would require that calibrations were performed for each subject if they were barefoot.

## Results and Discussion

**Figure 3:**
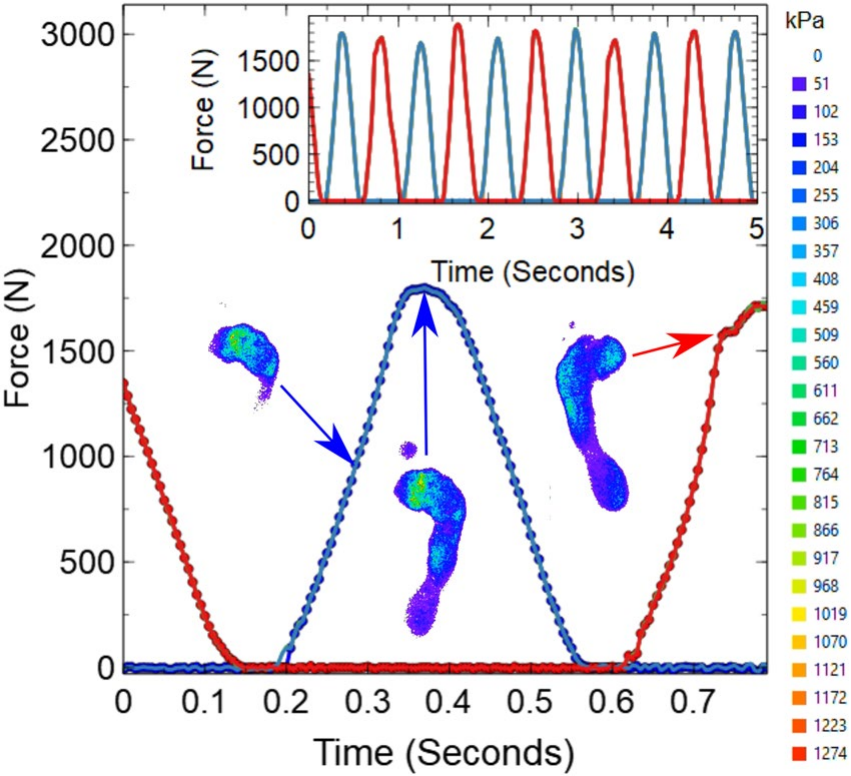
FTIR force data collected from a subject jogging on the spot barefoot. The main panel shows the variations in the total force applied to the platform under the left foot (blue) and the right foot (red) over a period of 0.8 seconds. The solid lines show data obtained using the load cells and the data points show the forces obtained by summing the pressures obtained from images similar to those shown in the insets. These images show the spatial variations in pressure beneath the feet at each time step (every 5 milliseconds). The legend on the far right of the figure gives the correspondence between colour and pressure in these images. The force-time curve shown in the inset shows the variation in the measured force over the entire duration of the experiment (5 seconds). A movie showing the evolution of the pressure distribution over the duration of the entire period is included in supplementary information (See SupplementaryMovie1.avi).

Figure 3 shows an example of data collected during an experiment where a subject was asked to jog on the spot for five seconds. During this time data were collected from both the load cells and by the camera at a rate 200 Hz (or frames per second). The data in the main panel shows a 0.8 second section of the data taken from a much longer run (see inset Fig. 3). The main panel in this figure demonstrates the high level of agreement between the forces obtained directly from the load cells and the forces obtained by summing the pressures obtained from the FTIR images (examples of these images are shown as insets in the main panel).

As expected, the measured force varied with time in a relatively uniform and consistent way as the weight of the subject was transferred from their left foot (blue data points and lines) to their right foot (red data points and lines). There is evidence in the data that the subject tended to favour their right foot (as shown by the larger peak forces in the inset). However, this total force data alone is unable to provide detailed information about how the pressure exerted beneath the subject’s feet was distributed at any given time. While simple force plates (based upon load cell measurements) provide the capability to determine the centre of pressure exerted by a given subject, they do not provide the means to determine the details of how pressure is distributed beneath a foot or a shoe outsole. The complimentary information that is given by the FTIR pressure map images shown as insets in Fig. 3 and in the movies provided in accompanying supplementary material provide an additional level of detail about where the pressure is being exerted and how high it is (See SupplementaryMovie1.avi).

**Figure 4:**
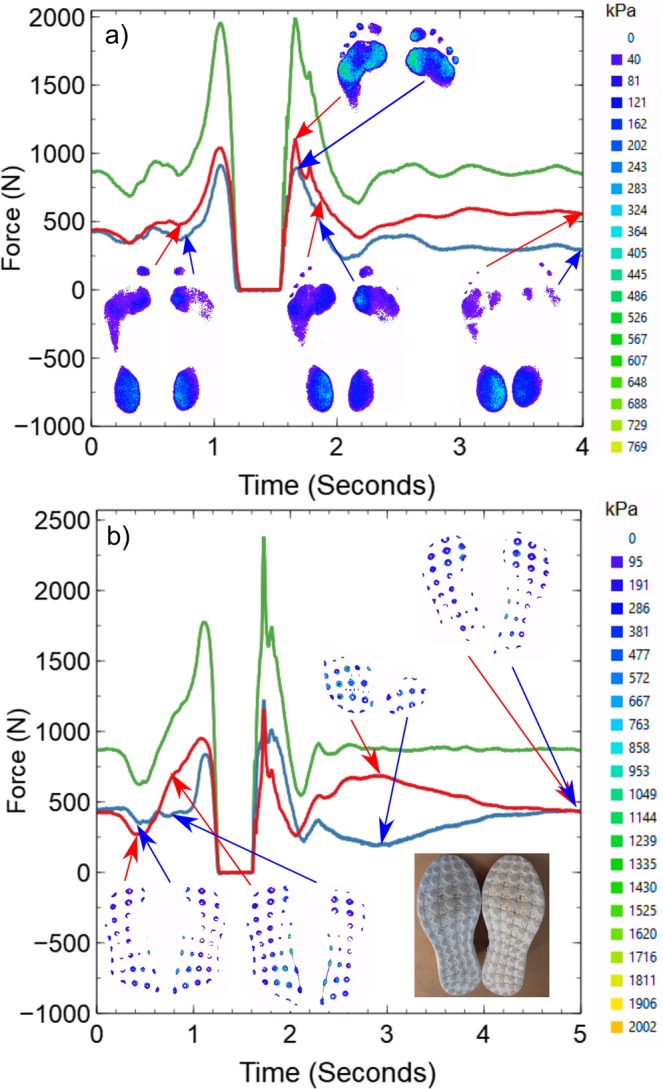
FTIR force data obtained during countermovement jumps. Data are shown for the force exerted on the platforms beneath the left (blue) and right (red) feet during a countermovement jump manoeuvre performed barefoot (panel a) and wearing shoes (panel b) both by the same subject. In both cases the force data are obtained by summing the pressures obtained from FTIR contact images (examples shown as insets). The total force exerted on both platforms (green) was obtained by summing the red and blue curves. The legends on the right hand side of the panels give the correspondence between the colour and pressure in the contact pressure map images shown as insets. The inset in panel b also shows a simple photograph of the tread pattern on the underside of the shoe. Examples of movies showing the variation in pressure over the entire duration of each experiment are provided as supplementary material (see SupplementaryMovie2a.avi and SupplementaryMovie2b.avi).

Figure 4 shows the results of two experiments where the same subject was asked to perform a simple countermovement jump both barefoot and while wearing shoes. A countermovement jump is a simple exercise that is used to build strength and develop explosive movements in athletes. The subject starts from a standing position with their feet slightly wider than shoulder width apart. They then move into a squatting position with as much of their weight in their heels as possible, before quickly jumping straight up, landing and controlling the deceleration. The data in both panels of Fig. 4 shows examples of force-time curves extracted from the two platforms beneath the left and right feet as well the sum of the two curves. In each panel the force data were obtained by summing the pressures obtained from the contact images (examples shown as insets) and were compared directly to the load cell measurements. The curves shown in this figure have the typical form expected for a force-time measurement obtained during a countermovement jump. The initial squat and take off phase occurs in the region of the curves below 1.2 seconds. This is followed by a region of zero force when the subject is not in contact with the platform (between 1.2 and 1.6 seconds) and then an impact and landing phase (greater than 1.6 seconds) where the subject finally comes to rest. In both cases, the weight of the subject could be found from their final resting force. In this case, the subject had a mass of approximately 86 kg (840 +− 10 N). Once again, the pressure maps shown in Fig. 4 give additional detail about how the forces are distributed beneath the subject at each time (see SupplementaryMovie2a.avi and SupplementaryMovie2b.avi). What is clear from both the force-time curves and the pressure map images is that the subject was favouring their right side when performing the jumps. In both panels of Fig. 4, the forces generated on the right side tend to be higher, but the contact images also show that the area of contact and the local pressures observed on the right side tend to be higher than on the left. At this point it is worth recalling that the contact regions are imaged from beneath so the images on the left are for the right foot (even though they look like a left foot/shoe print). Moreover, the images also show that the pressures are not distributed evenly under either of the contact objects and there are pressure hotspots in certain regions. For example, in the images shown in in both panels (a) and (b) the subject is clearly distributing their weight on the interior edges of the heel and mid-foot regions during take-off (approx. 0.8 seconds, panels a and b) and after landing (approx. 4 seconds, panel a). In panel (a) there is also evidence that the pressure is not distributed evenly within the forefoot region either between the feet or beneath a single foot. The additional information that is provided by the pressure map images therefore has great value in helping athletes to optimise their weight distribution and in providing coaching staff with a tool to monitor an individual’s progress during routine training activities.

**Figure 5:**
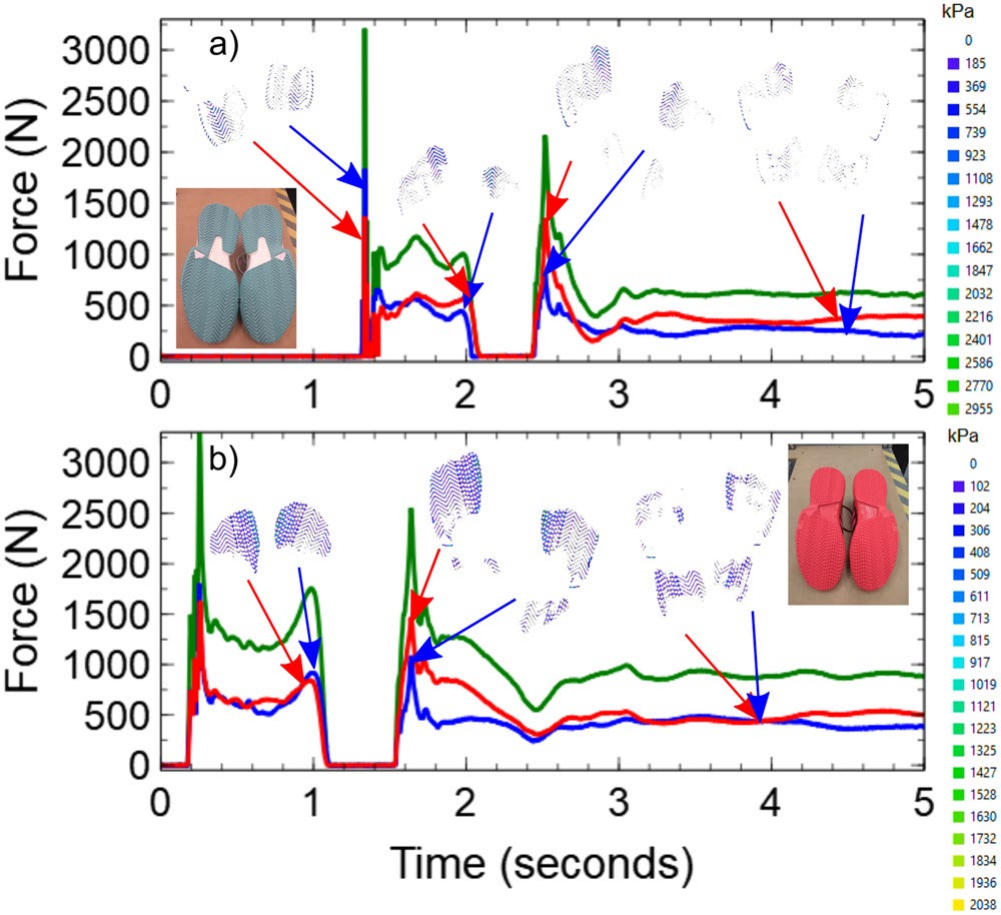
FTIR force data obtained during drop jumps. Data are shown for the force exerted on the platforms beneath the left (blue) and right (red) feet during a drop jump manoeuvre performed by a subject wearing shoes with blue outsoles (panel a) and a second subject wearing shoes with red outshoes (panel b). In both cases the force data are obtained by summing the pressures obtained from FTIR contact images (examples of which are shown as insets). The total force exerted on both platforms (green) was obtained by summing the red and blue curves. The legends on the right hand side of the panels give the correspondence between the colour and pressure in the contact pressure map images shown as insets. The inset in both panels also show simple photographs of the tread pattern on the underside of the shoes.

Figure 5 shows examples of force- time curves obtained from two individuals performing drop jumps. This manoeuvre involved the subject standing on a step (approximately 26 cm high), stepping/dropping off the step and landing in a squat position before jumping straight up, landing and coming to rest on the platform. The different phases of the manoeuvre can be identified in the force curves shown in panel a. The drop phase corresponds to the time below ~1.3 seconds. This is followed by the landing, squatting and jumping phase between 1.3 and 2 seconds. The second landing (after the jump) occurs at around 2.4 seconds before the subject finally comes to rest at a time of around 4 seconds. The plots for the second subject (shown in panel b of the same figure) contain many similar features but they occur at different times.

In addition to revealing differences in the weight and spatial pressure distributions beneath the two subjects during their respective manoeuvres, the pressure map images shown in Fig. 5 demonstrate that the different coloured shoe outsoles can be used to acquire FTIR image data. While the images obtained for the blue shoe outsoles tended to be more noisy than those obtained from the red shoe outsoles, it was still possible to obtain reliable information about the pressure distributions beneath them. The acquisition of higher quality data from the red outsoles is to be expected based on the fact that the LEDs used to excite the waveguide in this work were red (wavelength 632 nm). Sharp *et al*. have shown that the better an object is at scattering light of a particular wavelength, the less noisy the pressure-intensity response will be^11^. Optimisation of the image quality for a particular shoe outsole colour can therefore be achieved by simply changing the colour of the LEDs that are used to illuminate the waveguide. When attempts were made to use black and other dark coloured shoe outsoles (where visible wavelengths are not scattered very well) it was found that the FTIR technique did not work well and it was impossible to form useful pressure maps. However, these materials will often scatter other types of radiation and infrared (IR) LEDs can be used to excite the waveguide for these materials. When using IR LEDs, careful consideration may need to be given to the material used in constructing the waveguide imaging element in order to avoid absorption effects within the waveguide itself.

There are a few limitations of the FTIR imaging technique that should be taken into consideration when using it as a pressure measurement device. These are almost all centred around the uniformity of the mechanical and optical properties of the object being brought into contact with the waveguide surface. As Sharp and co-workers have demonstrated previously^11^, the force sensitivity of the light scattering response in FTIR imaging arises due to changes in the conformity of contact between the contacting object and the waveguide surface as well as the optical properties of the contacting material. FTIR measurements could therefore be expected to be influenced by factors such as changes in surface roughness of a contacting object, as well differences in colour and mechanical properties (e.g. Young’s modulus, Poisson’s ratio).

Changes in surface roughness have been shown to have very little effect on the FTIR response. This was found to be true for the contacting objects (shoes and feet) used in the present study, with no detectable dependence being observed on the surface roughness of the contacting materials. Previously this was interpreted as being due to differences in the areal number density of different sized asperities on contacting objects with dissimilar roughness values^11^. The idea is that smaller surface asperities will have a larger areal number density than larger ones. Even though the individual smaller asperities will have a reduced area of contact, their increased number compensates for this when the light scattering phenomenon is averaged over a large enough region of the contacting object. The net result is that changing the asperity size/roughness has no effect upon the measured light scattering intensity providing that an area of the surface very much larger than a single asperity is being averaged (as is the case here). This lack of dependence of the light scattering intensity upon the nature of the surface roughness is apparent in equation 1.

In contrast, changing the colour of the contacting object and/or its mechanical properties has been shown to influence the FTIR response. For example, evidence for the effects of changing the colour shoe outsoles were observed in the increased noise in the pressure maps shown in Fig. 5. The blue shoe outsoles scattered the red light that was used to excite the imaging platform more weakly than the red shoe outsoles. This had a significant effect upon the scaling factors that were used to obtain agreement between the load cell values and the optically derived forces. In addition, the uncertainties associated with the optically derived forces tended to be higher (+/−40 N) for the blue shoe outsoles than for the red shoe outsoles (+/−10 N).

The influence of both of the optical and mechanical properties is absorbed in the terms in the pre-factor in the intensity-pressure response given by equation 1. This equation can be rewritten in the form, ${I}_{pp}=\kappa {P}^{\frac{2}{3}}$, where,

2 $$\kappa =\frac{{\pi }^{4}{A}_{p}{I}_{o}{\alpha }^{2}(1+co{s}^{2}{\theta }_{s}){\varphi }_{o}}{2{\lambda }^{3}{D}^{2}{n}_{o}cos{\theta }_{r}}{[\frac{3(1-{\nu }^{2})}{E}]}^{\frac{2}{3}}$$

In particular, information about the optical properties of the contacting material can be found in the parameters *α* and *n*_*o*_, while the mechanical properties are captured by the Young’s modulus, *E* and Poisson ratio, *ν*. Having all of these parameters contained in the prefactor has the effect of preserving the overall shape (power law dependence) of the intensity-pressure response when the contacting object is changed. This means that if the new contacting object has uniform mechanical properties and it is uniform in colour, a simple calibration measurement can be performed to determine the new value of *κ*. However, potential difficulties arise if the contacting object has different coloured regions on its surface, or if it has regions with different mechanical properties. Under these conditions, each separate region with a different colour/elastic properties will have a different value of *κ* and will need to be treated separately. Any such heterogeneity in the properties of the contacting object will make interpretation of the data obtained using FTIR images difficult. For example, more sophisticated image processing algorithms would be needed to identify regions of different colours (e.g. on multi-coloured shoe outsoles) and process them separately in order to extract the correct force/pressure distributions. Identifying regions with different mechanical properties would prove significantly more challenging unless they also had different optical properties to neighbouring regions that would enable them to be identified more easily. It is worth noting that the white regions of the outsoles in the shoes used in Fig. 5a) did not enter into contact with the waveguide during the drop jump manoeuvres and therefore did not cause any issues related to non-uniformity of the properties of the outsoles. Had the white regions entered into conformal contact with the waveguide surface then more complicated analysis similar to that described above would have been required to extract the correct pressure distributions beneath theses outsoles.

In reality, the above limitations can be overcome by working with shoe outsoles of uniform colour and mechanical properties. This is something that can be done relatively easily in a clinical setting or in the development of new products by the use of shoes with outsoles that have uniform mechanical properties and a single colour. Bare human feet also have properties that do not vary significantly across their surface for any given individual- at least within the context of the present technique. However, experiments have shown that differences in the mechanical and optical properties of bare feet between individuals can be significant enough to justify that a new calibration of the optical forces be obtained for each new subject.

Another aspect of the work presented here that could potentially be viewed as a limitation relates to the use of load cells to perform the calibrations of the optically derived forces. However, the use of inexpensive load cells to provide a separate and independent measure of the total force exerted on the imaging platforms allows the user to gain additional confidence in the force measurements that are derived from the scattered light measurements. The internal checks that are also provided by the load cells during each measurement allow for any potential changes in the calibration of the instrument and/or changes in the material properties of a contacting object to be detected quickly. This would prevent the collection of erroneous data. In this sense, the inclusion of the load cells in the measurement system is a strength of the technique rather than a limitation.

In summary, we have shown that the FTIR imaging technique is useful for measuring pressure distributions beneath shoes and feet during a range of different dynamic contact experiments including jogging, countermovement jumps and drop jumps. However, the examples presented in Figs 3, 4 and 5 are not the only possible applications of the technique. A similar approach could be used to monitor the changing load distribution beneath athletes performing a range of other training activities^24^ or simply during standing. The device also has potential applications in physiotherapy for monitoring improvements during the rehabilitation of injured athletes or in elderly patients who may have suffered a fall. Measurements of the pressure distribution beneath patients with new prostheses and orthotic devices may also be valuable in improving a patient’s balance and comfort.

## Conclusions

FTIR imaging is a versatile technique for measuring the evolution of the pressure distribution beneath human feet and shoe outsoles. A simple device based upon an acrylic waveguide and a USB camera was shown to provide detailed information about the pressure distribution beneath these objects during a range of different movements including jogging, countermovement jumps and drop jumps. In each case, the forces obtained from the pressure maps that were extracted from the FTIR images were found to be in good agreement with those measured directly using load cells. The ability to provide high-resolution spatial information about the changing pressure distributions beneath shoes and feet at high frame rates means that this technique has potentially numerous applications in the treatment and monitoring of diseases/injuries of the foot and ankle, as well as having application in the design of footwear, prostheses and ortheses.

### Ethical Approval and Informed Consent

All experimental protocols were approved by the Faculty of Medicine and Health Science Ethics Committee at the University of Nottingham, UK (FMHSEC-UN, Reference number: 125–1707). Informed consent was obtained from all participants and all experiments were performed in accordance with the guidelines set out by the FMHSEC-UN.

## Supplementary information

Supplementary Movie 1

Supplementary Movie 2a

Supplementary Movie 2b

Supplementary information

## Data Availability

All data generated or analysed during this study are included in this published article.
