# Supplementary material for: Dual optical force plate for time resolved measurement of forces and pressure distributions beneath shoes and feet
Source: Sci Rep. 2019 Jun 20;9:8886. doi: 10.1038/s41598-019-45287-9 (PMC6586862; doi:10.1038/s41598-019-45287-9)
Supplement: Supplementary file 4 — Supplementary information [file 41598_2019_45287_MOESM4_ESM.docx]

**Dual optical force plate for time resolved measurement of forces and pressure distributions beneath shoes and feet**

Christopher G. Tompkins and James S. Sharp^*^

School of Physics and Astronomy, University of Nottingham, University Park, Nottingham, NG7 2RD, UK

*Corresponding Author, email: james.sharp@nottingham.ac.uk

**Supplementary Information**

**SupplementaryMovie1.avi** – A movie showing the evolution of pressure beneath a subject’s feet as they jogged on the spot. This movie was recorded at 200 frames per second and is played back at 30 frames per second. The inset shows the forces exerted on the plates beneath each foot (red=right foot, blue=left foot) and the sum of the forces from both panels (green).

**SupplementaryMovie2a.avi** – A movie showing the evolution of pressure beneath a subject’s feet as they performed a countermovement jump. This movie was recorded at 200 frames per second and is played back at 30 frames per second. The inset shows the forces exerted on the plates beneath each foot (red=right foot, blue=left foot) and the sum of the forces from both panels (green).

**SupplementaryMovie2b.avi** – A movie showing the evolution of pressure beneath a subject wearing sports shoes as they performed a countermovement jump. This movie was recorded at 200 frames per second and is played back at 30 frames per second. The inset shows the forces exerted on the plates beneath each foot (red=right foot, blue=left foot) and the sum of the forces from both panels (green).
